# Prevalence and burden of HBV–HIV co-morbidity: a global systematic review and meta-analysis

**DOI:** 10.3389/fpubh.2025.1565621

**Published:** 2025-04-04

**Authors:** Mequanente Dagnaw, Achenef Asmamaw Muche, Bisrat Misganaw Geremew, Lemma Derseh Gezie

**Affiliations:** ^1^Department of Epidemiology and Biostatistics, Institute of Public Health, University of Gondar, Gondar, Ethiopia; ^2^Department of Medical Biotechnology, Institute of Biotechnology, University of Gondar, Gondar, Ethiopia

**Keywords:** HBV, HIV, systematic review, meta-analysis, art, HBV-HIV comorbidity

## Abstract

**Introduction:**

Hepatitis B is a serious liver infection caused by the hepatitis B virus (HBV). Because of the shared modes of transmission, co-infections of HBV are common among people living with Human Immunodeficiency Virus (HIV) infection. While the use of antiretroviral therapy (ART) has significantly improved the life expectancy of HIV patients, hepatitis viral co-infections have become increasingly important. Particularly, HBV infection remains under-diagnosed and under-reported, despite its highly infectious nature. Therefore, this review was aimed at understanding the burden of hepatitis B disease among adults living with HIV receiving ART.

**Methods:**

Using pertinent search terms, all research found in Google Scholar, HINARI, EMBAS, Scopus, and PubMed was located. Data were extracted following the evaluation of the evidence using the Joanna Briggs Institute’s cross-sectional and cohort study methodologies.

**Result:**

A total of 18 groups involving 71,411 adults with HBV–HIV were selected for the study. Of those, 10.21% with 95% CI (5.06, 15.36) and 11.05% with 95% CI (2.78, 19.32) of HBV–HIV adults worldwide had an overall prevalence of HBV, with an I^2^ value of 0.0% (*p*-value = 0.729) and an I^2^ value of 0.0% (*p*-value = 0.818) from cross-sectional and cohort studies, respectively.

**Conclusion:**

The global prevalence of people living with HBV–HIV is high, which poses a serious risk to public health. The review can clearly show the current pooled prevalence of HIV–HBV in the world, which may be helpful for policymakers because a large number of recent studies were included in it. Thus, it is strongly advised to broaden the current preventive and control program’s purview and implement new, sensitive screening, testing, and treatment techniques. To raise community awareness, it would also be preferable to revamp the current prevention and control program and establish target-specific task forces at various health facility levels.

## 1 Introduction

Chronic Hepatitis B (CHB) infection is a long-term liver infection caused by the hepatitis B virus (HBV). It is a major public health problem, resulting in an estimated 820,000 annual deaths in 2023. Although HBV can be prevented with vaccination, an estimated 296 million people in 2024 will be living with Chronic Hepatitis B (CHB) (1, 2). According to the World Health Organization (WHO), 1.1 million people die each year, and 3.0 million new infections are caused by HBV (3). Chronic hepatitis B (CHB) causes liver cancer, cirrhosis, chronic liver infection, and hepatic failure, leading to death (4). The Western Pacific and African regions have the highest infection loads, with 116 million and 81 million chronically infected people, respectively. Around 60 million people are infected in the Eastern Mediterranean region, 18 million in the Southeast Asia region, 14 million in the European region, and 5 million in the Americas (5). Almost 80% of nations, including every nation in Africa, have declared viral hepatitis to be a serious public health emergency (6). Sub-Saharan Africa (SSA) and the West Pacific region account for 79% of chronic cases of HBV (7). The majority of the persons who become chronic carriers of HBV live in Asia and Africa.

Even though PLHIV live longer due to increased access to antiretroviral therapies, they are still susceptible to opportunistic infections and co-infections, particularly with the hepatitis B virus, as a result of different reasons (8). People living with HIV infection are susceptible to co-infection with HBV, particularly when the virus progresses to the AIDS stage (9). This is because they share comparable modes of transmission, such as risky sexual behavior, mother-to-child transmission, sharing sharp objects, sharing intravenous drugs, and giving blood contaminated with HIV and hepatitis (10). Additionally, an HIV infection may lower the host’s immunity, which could result in the reactivation of the hepatitis virus. Furthermore, liver disease may worsen because of the side effects of HIV drugs. Therefore, compared to individuals who are not co-infected, those who have HBV/HIV co-infection are more likely to die (11). Rapid mortality is attributed to hematological problems, organ failure, and chronic liver disease.

Globally, an estimated 5%–30% of people living with HIV (PLHIV) are co-infected with HBV (5). Co-infection is 5%–10% common in North America, Europe, and Australia, and 20%–30% common in Asia and sub-Saharan Africa (SSA) (6, 7). In 2023, a total of 39.9 million people were infected with HIV, of whom 5%–10% of people were co-infected with HBV (12). Over 70% of HIV cases worldwide are found in sub-Saharan Africa, where HBV is common and causes the majority of virally related chronic liver disease disorders (12). Consequently, approximately 71% of HIV/HBV-co-infected individuals reside in sub-Saharan Africa.

For people living with HIV/AIDS, chronic hepatitis B (CHB) infections are the main cause of liver disease complications and acceleration to advanced stages, such as cirrhosis and hepatocellular carcinoma (HCC) (13, 14). The natural course of hepatitis B infection is significantly affected by HIV infection. Most of the time, HIV infection’s suppression of the immune system promotes hepatitis B replication in hepatocytes, which in turn exacerbates chronic liver disease sequelae, including liver cirrhosis and fibrosis (13–15). Compared to HBV mono-infection, the coexistence of HIV and hepatitis B viruses increases the chronicity, early development, and high mortality of liver disorders (16).

There is a need to establish the updated global burden of HIV–HBV co-infection among ART followers, to characterize the most affected populations and geographical regions, and to inform national and regional screening programs and clinical management. However, to date, only one review has estimated the global burden of HBV co-infection among patients with PLHIV, which lasts 6 years. Existing estimates suggest that approximately 7.6% of patients with PLHIV have chronic hepatitis B or 2.7 million people, but these estimates were based on small numbers of studies with unclear methodology. We, therefore, undertook a global systematic review of the prevalence and burden of HBsAg in patients with PLHIV.

## 2 Methods and materials

### 2.1 Protocol and registration

The review methodology was based on the Preferred Reporting Items for Systematic Reviews and Meta-Analyses (PRISMA) 2020 checklist (17).

### 2.2 Eligibility criteria

The evaluation comprised primary investigations of observational studies (cross-sectional and cohort) aimed at determining the global prevalence of hepatitis B patients among adults with PLHIV on ART and/or risk factors. The included studies were those carried out at the facility level. Enrollment was open to all papers published in English up to 20 April 2024, with no restrictions on the study period. Any adult (≥15 years old) co-infected with HIV or HBV who was receiving ART was eligible to participate in the study. The primary target audience for this exposure was ART-using HIV-positive individuals. The incidence or prevalence of hepatitis B virus or hepatitis B virus risk factors in individuals co-infected with HIV and HBV is the outcome of interest.

### 2.3 Exclusion criteria

Research that met any of the enumerated criteria was disqualified: We excluded the following from the review: abstracts, editorial reports, letters, reviews, and commentaries, even after contacting the corresponding author(s); articles lacking a full text and requiring significant effort to extract data; studies that solely reported qualitative findings; we only took into account the quantitative findings in studies that reported both quantitative and qualitative findings; we only took into consideration the HBV findings in studies that reported both HBV and HCV results; and studies with methodological flaws, such as improper outcome ascertainment criteria.

### 2.4 Information source

To prevent duplications, databases were primarily searched for systematic reviews. A literature search technique was used to identify primary studies that have been published worldwide up until 20 April 2024 on the prevalence of hepatitis B and the variables that influence adult HIV-positive individuals receiving antiretroviral therapy.

Using a typical search method, published publications were obtained from major databases like PubMed, EMBASE, Scopus, and HINARI. Furthermore, gray literature published on Google Scholar was considered. In addition, authors were alerted via email of works that had been published, but they were not given free access to download and examine the entire manuscript. Additionally, a manual search of the publications that were part of the review was conducted.

The following terms or search strings were used in Medline PubMed: HIV/HBV, HIV/AIDS, Adult, Factors, Determinants, and Associated Factors; Prevalence; Magnitude; Hepatitis B; Hepatitis B infection; HIV/HBV comorbidity. The combined search phrases were used to find pertinent material tailored to the particular database’s requirements. Using the “Medical Subject Headings (MeSH)” and “All fields” and connecting the necessary Boolean operator terms (“AND” and “OR”), the search strategy was constructed in the advanced search databases based on the terms described above.

### 2.5 Search strategy

Six databases, namely PubMed, EMBASE, Scopus, HINARI, Advanced Google Search, and Google Scholar, were searched for relevant articles from inception to the current date (30 April 2024). The research question of the systematic review was clearly defined in terms of populations, interventions, comparators, outcomes, and study designs (PICOS). The search terms used were as follows: “HIV OR human immunodeficiency virus” and “hepatitis-B OR HBV” and “prevalen* OR inciden* OR seroprevalen* OR screening OR surveillance OR population* OR survey* OR epidem* OR data collection OR population sample* OR community survey* OR cohort OR cross-sectional OR longitud* OR follow-up.” The search queries were tailored to the search functionality of each database. The reference lists of the articles identified as reviews were screened for relevant sources.

### 2.6 Study screening and selection processes

Using the title and abstract, ZW and AJ, two impartial reviewers, filtered the publications. Then, two impartial reviewers examined the manuscripts that qualified for full-text review to determine whether to include them in a systematic review and meta-analysis. In each case where two impartial reviewers declined to include an article during screening or full-text review, a lead investigator was contacted.

Initially, duplicates were eliminated from the articles downloaded into the EndNote collection from databases and electronic search engines. Second, the topic, study participants, language, and study area are considered when evaluating the remaining papers. Third, papers with documentation in languages other than English and unrelated subjects were excluded. Finally, in order to determine the final article included, the full texts and abstracts of the remaining research were thoroughly scrutinized.

### 2.7 Outcome measurement and prioritization

Data extraction was performed following a thorough examination of measurement results. The prevalence of hepatitis B among adult HIV-positive individuals receiving ART was the review’s main focus. The factors used in primary studies were defined and considered. When independent factors and outcome definitions differed, the data were compiled and used for subgroup analysis to identify potential heterogeneity.

### 2.8 Patient and public involvement

This review’s main goal was to examine and compile the results of earlier research projects conducted by different academics. Consequently, neither the patients nor the patient advisors participated in the research.

### 2.9 Risk of bias and quality assessment

ZW and AJ, two separate reviewers, assessed the quality of the work. The two reviewers independently offered “High” and “Low” categories of quality ratings based on a set of criteria, which were then used to build a two-by-two contingency table and calculate “K.” The Agency for Healthcare Research and Quality (AHRQ) checklist and the standardized Joana Brig’s Institute (JBI) were used to evaluate the quality of each publication for cross-sectional and cohort studies, respectively (5, 6). An instrument with eight question items for analytical cross-sectional studies and 11 questions for cohort studies was used.

Each AHRQ checklist contains 3 alternative scores: Y=YES, N=NO, an item scores “1” if the answer is “YES,” while “0” if the answer is “NO” or “UNCLEAR” and Article quality is assessed as follows: low quality = 0–3; moderate quality = 4–7; high quality =8–11 and Each JBI Q1–Q11 indicates questions 1 to 11 based on the JBI risk assessment. The risk of bias was ranked as high when the study reached up to 49% of “yes” scores, moderate when the study reached 50 to 69% of “yes” scores, and low when the study reached more than 70% of “yes” scores. “✓” indicates yes, “✕” indicates no, and “?” indicates unclear. Despite the detailed review, the reviewers’ average scores were calculated for persistent disagreement. Similarly, each factor and outcome variable were critically appraised for determinants. A similar cutoff point used in the prevalence studies was applied for the factors. Moreover, the quality results of the primary studies were placed in a separate column in the data extraction form (Tables 1, 2).

**Table 1:** Quality assessment for each included cross-sectional study based on the checklist recommended by the Agency for Healthcare Research and Quality (AHRQ).

Included cross-sectional studies	The quality assessment for each study is based on a checklist recommended by the Agency for Healthcare Research and Quality (AHRQ)																							
	Q1		Q2		Q3		Q4		Q5		Q6		Q7		Q8		Q9		Q10		Q11		Total	Overall appraisal
	R1	R2	R1	R2	R1	R2	R1	R2	R1	R2	R1	R2	R1	R2	R1	R2	R1	R2	R1	R2	R1	R2		
1. Seyoum E. et al. (48)	1	1	1	1	1	1	1	1	1	1	0	0	1	1	1	1	1	1	1	1	1	1	10	High
2. Belayneh F et al. (11)	1	1	1	1	1	1	1	1	1	1	1	1	1	1	1	0	1	1	1	0	1	1	9	High
3. Weldemhret L. et al. (35)	1	1	1	1	1	1	1	1	1	0	0	1	1	1	1	1	1	0	1	1	0	1	7	Moderate
4. Denue BA. et al. (49)	1	1	0	0	1	1	1	1	0	1	1	1	1	0	1	1	1	1	0	1	1	1	7	Moderate
5. Attia KA. et al. (50)	1	1	1	1	1	1	1	1	0	0	0	0	1	1	1	1	1	1	1	0	1	1	8	High
6. Kellerman SE. et al. (51)	1	1	1	0	1	1	1	1	1	1	0	0	1	1	1	1	1	1	0	1	1	1	8	High
7. Martins S. et al. (52)	1	0	1	1	1	1	1	1	0	1	1	0	1	1	1	1	0	1	1	1	1	1	7	Moderate
8. Kye-Duodu G. et al. (53)	1	1	1	1	1	1	1	1	0	0	0	0	1	1	1	1	1	1	1	0	1	1	8	High
9. Bigna JJ. et al. (54)	1	1	1	1	1	1	1	1	1	1	0	0	1	1	1	1	1	1	1	1	1	1	10	High
10. Hailu D. et al. (55)	1	1	1	1	1	1	1	1	0	0	1	0	1	1	1	1	1	1	1	0	1	0	7	Moderate
11. Pappoe F. et al. (56)	1	1	1	1	1	1	1	1	0	0	0	0	1	1	1	1	1	1	1	1	1	1	9	High
12. Yemanebrhane N. et al. (57)	1	1	1	1	1	1	1	1	1	0	0	0	1	1	1	1	1	1	1	0	1	1	8	High
13. Matthews PC. et al. (58)	1	1	1	1	1	1	1	1	0	0	0	0	1	1	1	1	1	1	1	1	1	1	9	High

Y=YES, N=NO, an item scores “1” if the answer is “YES,” while “0” the answer is “NO” or “UNCLEAR” and article quality is assessed as follows: low quality = 0–3; moderate quality = 4–7; high quality = 8–11.

**Table 2:** Quality assessment for each included cohort study based on the checklist recommended by JBI risk of bias quality assessment criteria.

Included cohort studies	JBI risk of bias quality assessment for cohort studies												
	Q1^a^	Q2^a^	Q3^a^	Q4^a^	Q5^a^	Q6^a^	Q7^a^	Q8^a^	Q9^a^	Q10^a^	Q11^a^	% Yes	Risk^b^
1. Goa A. et al. (21)	✕	✓	✕	✕	✕	✕	✓	✕	✕	✕	✓	27.3%	High
2. Hawkins C. et al. (59)	✓	✕	✕	✓	✕	✓	✓	✕	✓	✓	✓	63.63%	Moderate
3. Msomi N. et al. (60)	✓	✓	✓	✕	✕	✕	✓	✓	?	✕	✕	45.45%	High
4. Nyirenda M. et al. (61)	✕	✓	✕	✕	✕	✕	✓	✕	✕	✕	✓	27.3%	High
5. Day SL. et al. (62)	✓	?	✕	✓	✕	✓	✕	✓	✕	✕	✕	36.36%	High

JBI, Joanna Briggs Institute.

a Q1–Q11 indicate questions 1 to 11 based on the JBI risk assessment.

b The risk of bias was ranked as high when the study reached up to 49% of “yes” scores, moderate when the study reached 50 to 69% of “yes” scores, and low when the study reached more than 70% of “yes” scores.

“✓” indicates yes, “✕” indicates no, and “?” indicates unclear.

### 2.10 Data extraction process

Following the identification of potentially eligible studies, the entire text of these studies was obtained, and two independent reviewers (AJ and ZW) separately evaluated each study’s eligibility. They had discussions to settle any differences regarding which studies should be included. Ultimately, the template was constructed to extract pertinent data from a Microsoft Excel spreadsheet. The summary table includes a list of items about study characteristics, such as the name of the primary author(s), participant age, study year, design, findings, sample size, study subjects, study setting, response rate, odds ratio, publication year, data collection method, and relevant factors associated with HBV prevalence that were extracted.

For quantitative studies, the prevalence of hepatitis B among patients with PLHIV who were on ART, the logarithm of the prevalence, and the standard error of the logarithm of the prevalence were computed. Likewise, for determinants, the odds ratio, logarithms of the odds ratio, and the standard error of the logarithms of the odds ratio were calculated. For any difficulties encountered during data extraction, the corresponding author(s) was/were contacted by any means of communication.

### 2.11 Outcome variable

Prevalence or incidence of hepatitis B among PLHIV on ART in the world.

The prevalence or incidence of hepatitis B among PLWH that are on ART in the world.

### 2.12 Data analysis, synthesis, and assessment of publication bias

A Microsoft Excel spreadsheet was used to extract data. The extracted data were exported to STATA software version 17 for further analysis. Tables, figures, and forest plots were used to describe and summarize the major investigations. The fixed effects and random-effects models for the pooled prevalence of HIV–HBV were used. Galbraith plots and Higgins I-Squared (I^2^) statistics have been used to evaluate heterogeneity. Sensitivity analysis and meta-regression were used to investigate the causes of heterogeneity. A funnel plot and Egger’s regression tests were used to determine publication bias, and trim and fill analyses were performed to treat publication bias. The odds ratio with a 95% confidence interval was used to quantify the measure of association for factors that affect the prevalence of hepatitis B among patients with PLHIV who are on ART worldwide. The existence of heterogeneity among studies was examined using the forest plot and I^2^ heterogeneity test (7), adopting a 50% standard as recommended by Cochrane guidelines (12). The I^2^ values of 25, 50, and 75% were interpreted as the presence of low, medium, and high heterogeneities, respectively. I^2^ heterogeneity test of ≥50% and a *p*-value of <0.05 was assured as the presence of heterogeneity. To identify influential studies that resulted in variation, sensitivity analysis was carried out using the “metaninf” command (18). Then, for extreme outlier study(s), the extracted data were checked for any errors that might occur during the process of extraction. Finally, the article(s) were excluded from the analysis if the data were error-free. Similarly, subgroup analyses were employed by assuming country, study design, and year of the study as grouping variables and sources of variation.

Using the “metafunnel” command (19) and objectively by Egger’s regression test, publication bias was detected (19). Accordingly, the funnel plot asymmetry and/or statistical significance of Egger’s regression test (*p*-value <0.05) suggest publication bias. Therefore, using the “metatrim” command, a nonparametric trim and fill analysis method was done (20).

Using the Laird random-effects model, the pooled prevalence of intention toward the prevalence of hepatitis B among patients with PLHIV on ART was reported. The association between the determinants and the pooled proportion of intention toward the prevalence of hepatitis B among patients with PLHIV on ART was estimated based on the effect size. Furthermore, all statistical interpretations were based on 95% CIs.

## 3 Ethics and dissemination

Ethical clearance was not a concern since this was a systematic review and meta-analysis. The results will be published in a reputable peer-reviewed journal and presented at scientific research conferences.

## 4 Results

### 4.1 Search results

**Figure 1:**
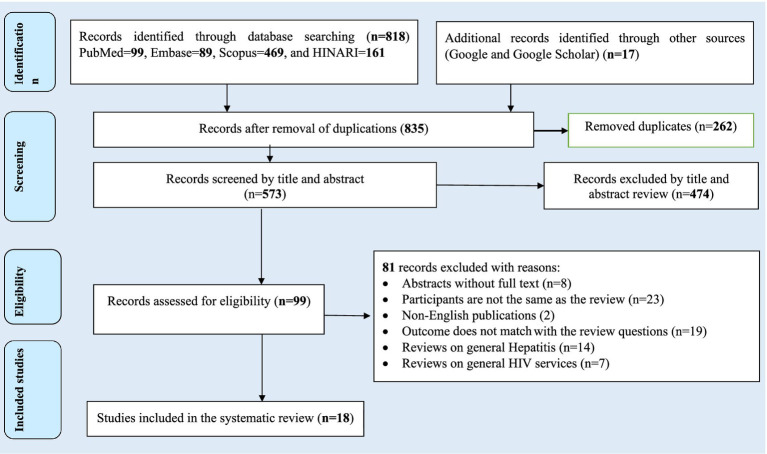
Flow chart of study selection for systematic review and meta-analysis of prevalence and burden of HBV co-morbidity among people living with HIV, 2024.

The search strategy resulted in 835 records through (PubMed = 99, EMBASE = 89, Scopus = 469, and HINARI = 161) databases. In addition, 17 studies were accessed manually using the Google and Google Scholar search engines. From these, 262 duplicated records were excluded, and from articles screened using their titles and abstracts, 474 were excluded. Therefore, 99 articles were assessed for eligibility. From these, 81 articles were excluded: 8 were abstracts without full text, 25 studies’ participants were not the same as the review, 19 studies’ outcomes did not match with the review questions, 14 studies were reviews on general hepatitis, and 8 studies focused on general HIV services review. Finally, 18 studies were included in the review (Figure 1).

### 4.2 Methodological quality of the included studies

The AHRQ and JBI quality appraisal criteria established for the cross-sectional and cohort studies were used, respectively. All studies presented clear research questions and collected data to address all questions, and the studies had representative samples and used appropriate statistical analysis. The studies included in this systematic review and meta-analysis had no considerable risk (had low risk). Therefore, all studies were considered to be included in the review.

### 4.3 Characteristics of the included studies

Twelve (66.67%) of the primary studies we found were published between 2015 and 2024; the other 9 (33.33%) primary studies included in the review were published between 2003 and 2014. A cross-sectional design was used in 13 (72.22%) of the studies included in the review, while 8 (27.78%) of the studies used a cohort design (Table 3).

**Table 3:** Description of primary studies included in the systematic review and meta-analysis of prevalence and burden of HBV co-morbidity among people living with HIV, 2024.

First author name	Publication year	Country	Study design	Participants	Age	Sample size	Prevalence
Goa A. et al. (21)	2019	Ethiopia	Cross-sectional	Adult HIV-positive	15–45	442	8.37
Seyoum E. et al. (48)	2022	Ethiopia	Cross-sectional	Adult HIV-positive	15–60	873	5.96
Belayneh F. et al. (11)	2015	Ethiopia	Cross-sectional	Adults living with HIV	18–45	348	6.90
Weldemhret L. et al. (35)	2016	Ethiopia	Cross-sectional	HIV/AIDS positive	18–45	508	5.91
Denue BA. et al. (49)	2012	Nigeria	Cross-sectional	HIV-infected patients	18–81	569	5.91
Attia KA. et al. (50)	2012	Cote d’Ivoire	Cross-sectional	HIV patients	18–66	608	3.45
Kellerman SE. et al. (51)	2003	Atlanta	Cohort study	HIV-infected patients	18–60	16,248	1.94
Martins S. et al. (52)	2014	Brazil	Cross-sectional	HIV-seropositive	18–81	300	2.33
Kye-Duodu G. et al. (53)	2016	Ghana	Cross-sectional	PLHIV	18–51	320	8.75
Bigna JJ. et al. (54)	2017	Cameroon	Cross-sectional	With HIV infection	NR	23,295	0.086
Hawkins C. et al. (59)	2013	Tanzania	Observational	Adults HIV-infected	31–44	17,539	6.15
Hailu D. et al. (55)	2022	Ethiopia	Cross-sectional	HIV-positive adults	24–65	300	9.67
Msomi N. et al. (60)	2020	South Africa	Cohort study	HIV-infected patients	24–35	4,292	8.46
Pappoe F. et al. (56)	2019	Ghana	Cross-sectional	HIV positive	19–60	394	6.09
Yemanebrhane N. et al. (57)	2017	Ethiopia	Cross-sectional	HIV/AIDS-infected	18–50	384	4.69
Matthews PC. et al. (58)	2015	Botswana	Cross-sectional	HIV-1 positive women	NR	950	7.58
Nyirenda M. et al. (61)	2008	Malawi	Observational	HIV-infected inpatient	18–80	226	12.83
Day SL. et al. (62)	2013	Kenya	Cohort study	HIV-1-positive	18–64	159	6.92

**Figure 2:**
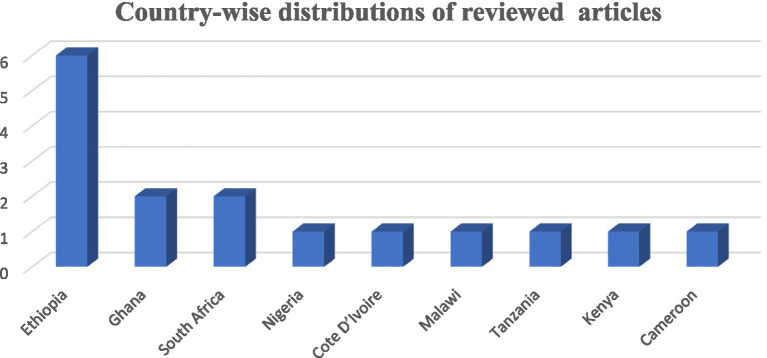
Continental distributions of the study of prevalence and burden of HBV co-morbidity among people living with HIV, 2024.

In the majority of these studies 16 (88.89%) were carried out on the African continent, and two (11.11%) were carried out on the African continent. Among those that were done on the African continent, 6 (37.5%) were carried out in Ethiopia, 2 (12.5%) were in Ghana, 2 (12.5%) were in South Africa, 1 (6.25%) was in Nigeria, 1 (6.25%) was in Cote d’Ivoire, 1 (6.25%) was in Malawi, and 1 (6.25%) was in Tanzania, while the other two were in Kenya (6.25%) and Cameroon (6.25%) (Figure 2).

A total of 71,411 study participants were estimated to participate in all the studies. The sample size of individual primary studies ranged from 159 in Kenya to 23,295 in Cameroon.

In the majority of the studies reviewed, the prevalence of HBV–HIV comorbidity was substantially high (21). In the reviewed studies, the prevalence of HBV among patients with PLHIV who are on ART was 31.8% in Cote d’Ivoire and 2.3% in Brazil. The study setting for all included studies was facility-based, and all study participants were PLHIV patients on ART.

## 5 Meta-analysis

### 5.1 A Galbraith plot test

**Figure 3:**
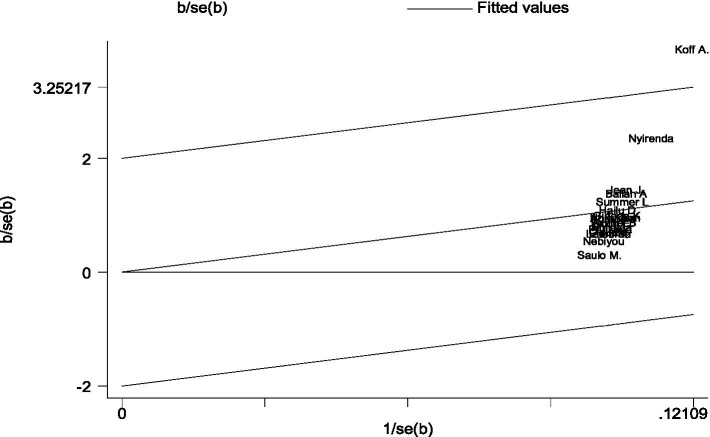
A Galbraith plot of articles included in the review to look for the existence of heterogeneity from cross-sectional studies.

The Galbraith plot was also used to assess heterogeneity and detect potential outliers. In the absence of substantial heterogeneity, we expect around 95% of the studies to lie within the 95% CI region: Hence, 1 out of the 13 cross-sectional studies was outside the 95% CI region, which indicates considerable heterogeneity among the effect sizes. One study lies far away from the 95% CI region considered as outliers (22) (Figure 3).

**Figure 4:**
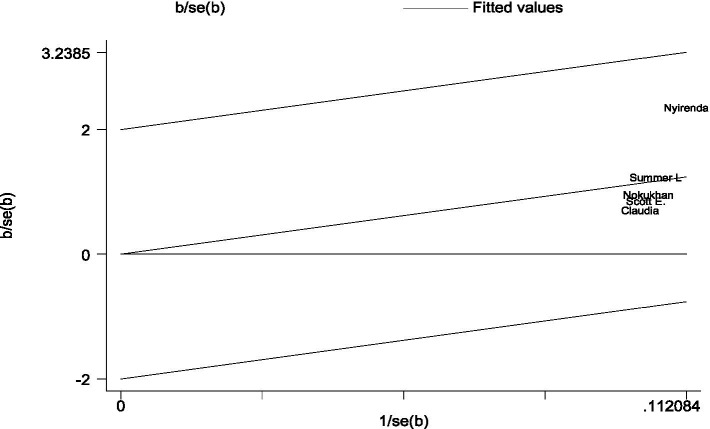
A Galbraith plot of articles included in the review to look for the existence of heterogeneity from cohort studies.

There are no cohort studies that were outside the 95% CI region, which indicates there is no considerable heterogeneity among the effect sizes (22) (Figure 4).

### 5.2 Pooled prevalence of people living with HIV–HBV comorbidity

In a meta-analysis, the summary effect is typically estimated using two different types of models. These models, which include fixed and random effects, each have their own set of presumptions. The summary effect is an estimation of the common effect size in a fixed effect model, which implies that the true effect size is the same across all studies (sampling error is the only reason for variability) (23). According to a random-effects model, which also assumes that the true effect size varies from study to study, the studies included in the analysis constitute a random sampling of effect sizes that could have been observed in each study. Our estimation of the mean of these effects is the summary effect (variability of the effect sizes is due to systematic error) (24).

As can be seen from the forest plot, the existence of high heterogeneity between included studies, which could be explained by I^2^ = 0.0% (*p* = 0.729) of the variation in effect sizes, is not due to between-study heterogeneity caused by sampling error at *p* > 0.05. Therefore, no need for a random-effects model has a mechanism to handle this kind of variability; therefore, this review did not employ a random-effects model to combine the prevalence of HIV–HBV comorbidity in the world.

**Figure 5:**
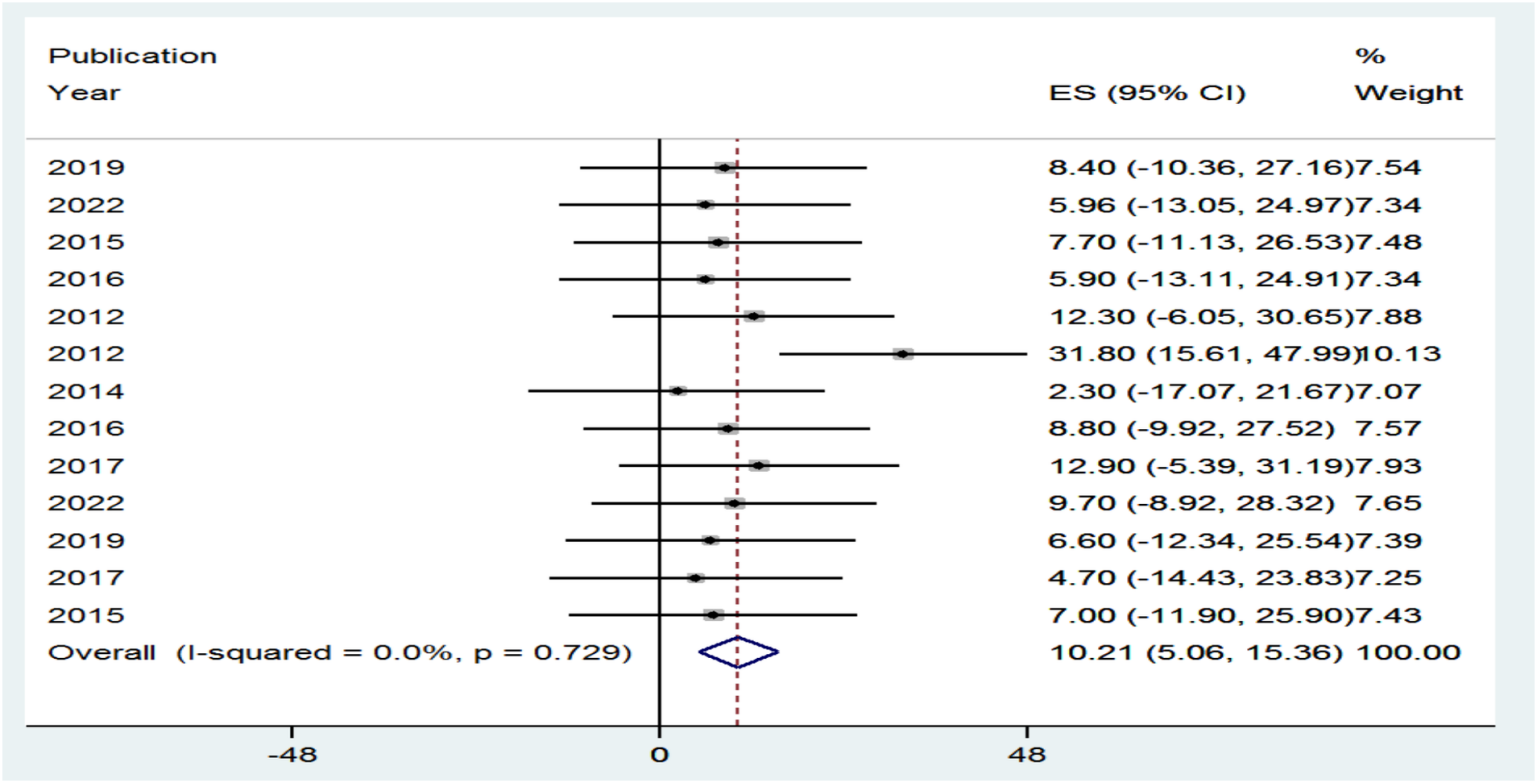
Forest plot showing the pooled prevalence and burden of HBV co-morbidity among people living with HIV in the world from cross-sectional studies, 2024.

**Figure 6:**
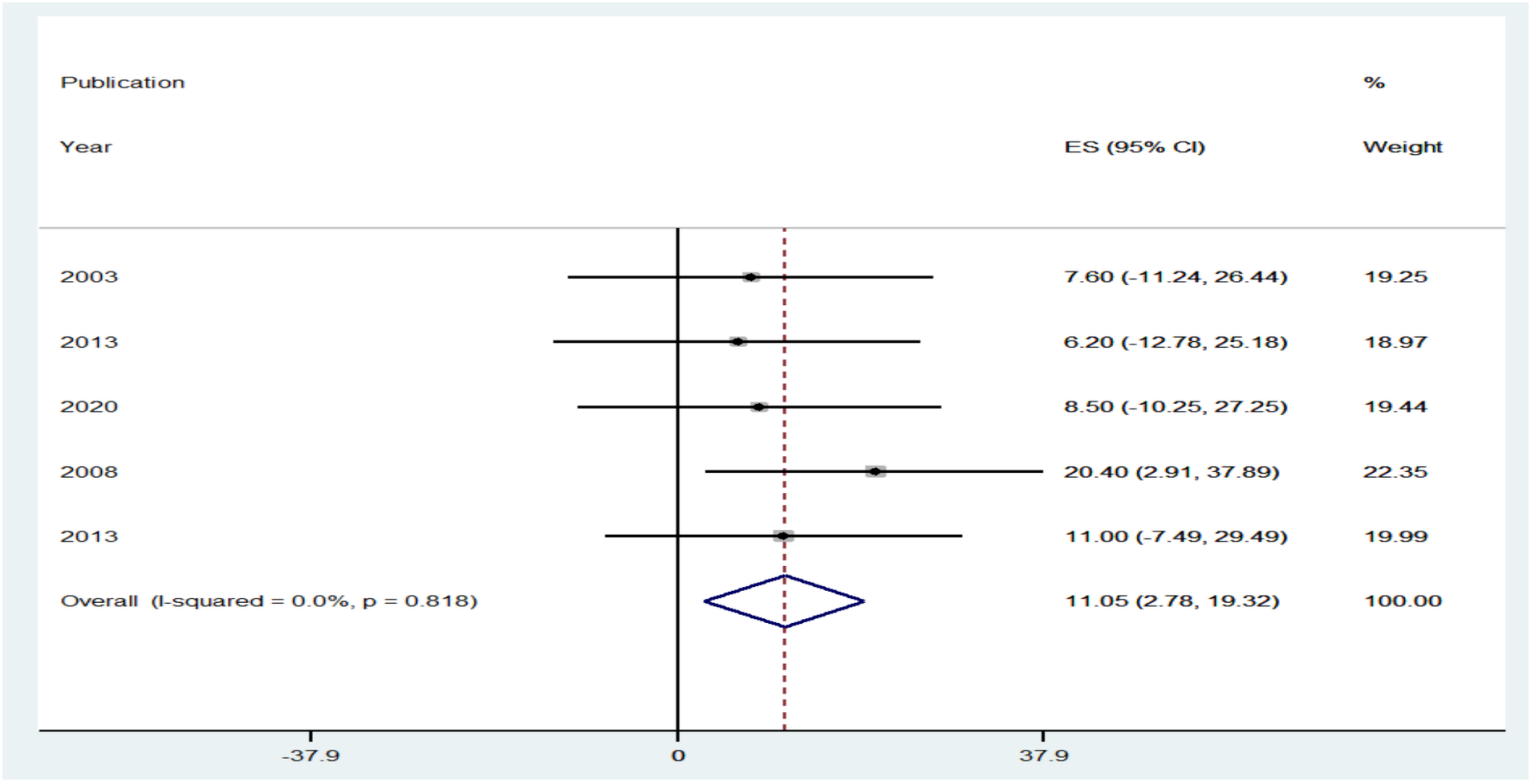
Forest plot showing the pooled prevalence and burden of HBV co-morbidity among people living with HIV in the world from cohort studies, 2024.

The prevalence of PLHBV–HIV comorbidity in the primary study ranged from 2.3 to 31.8%. The pooled prevalence of HIV–HBV comorbidity was found to be 10.21 and 11.05% from cross-sectional and cohort studies, respectively (Figures 5, 6).

### 5.3 Subgroup analysis

The prevalence of PLHIV–HBV was highly influenced by different risk factors in primary publications included in this review. The authors hypothesized that study country, study setting, publication year, and study period might be the sources of the high heterogeneity between studies included in the review, even though this was not confirmed in the forest plot. In order to determine the most likely reason for heterogeneity, subgroup analysis was conducted by dividing the effect sizes by study country, study setting, publication year, and study period. The effect size did not show a statistically significant subgroup effect for study country, study setting, publication year, and study period at *p* > 0.05, according to the subgroup outcome.

**Figure 7:**
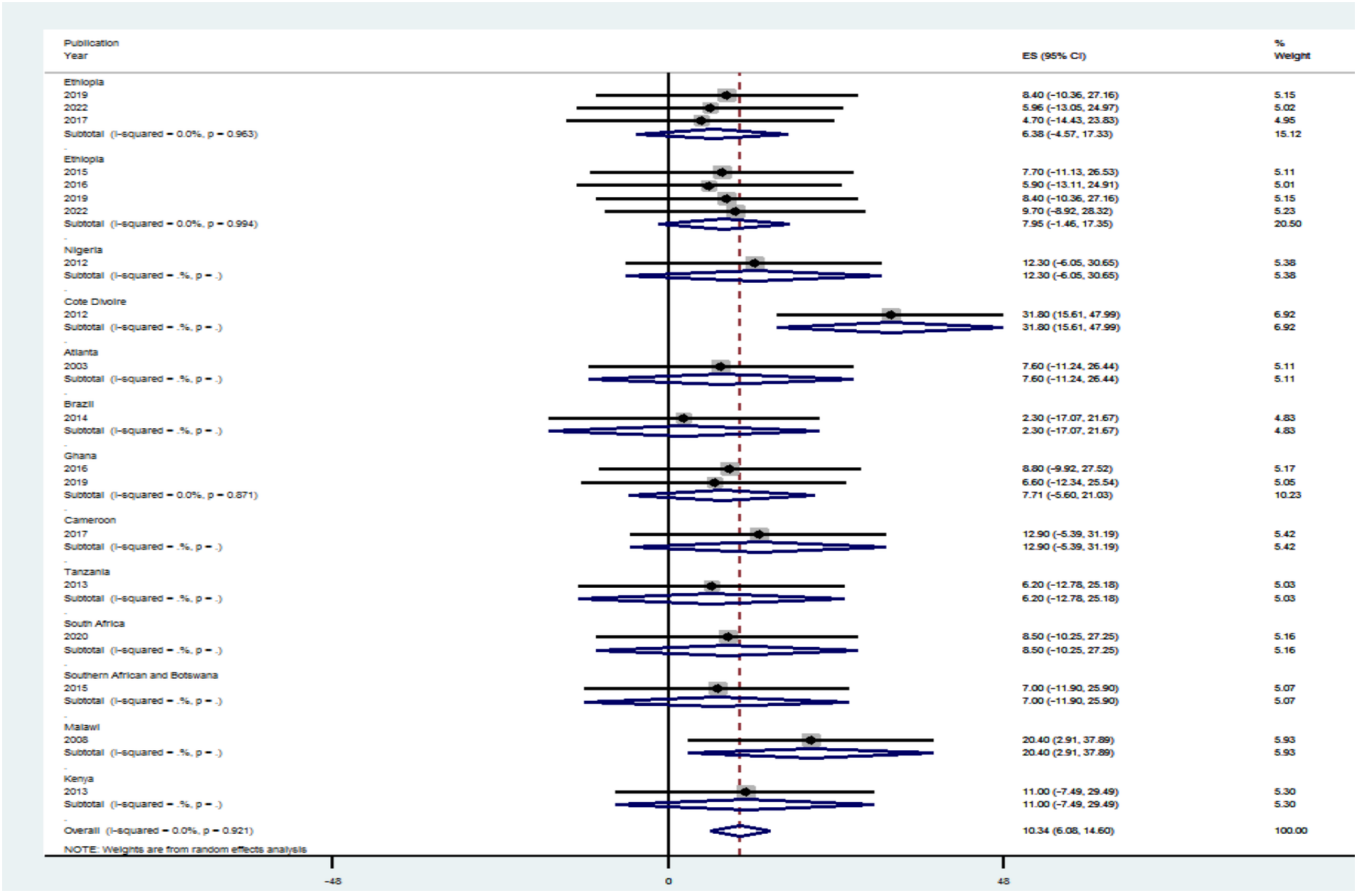
A graphical inspection of subgroup analysis from cross-sectional studies by country.

**Figure 8:**
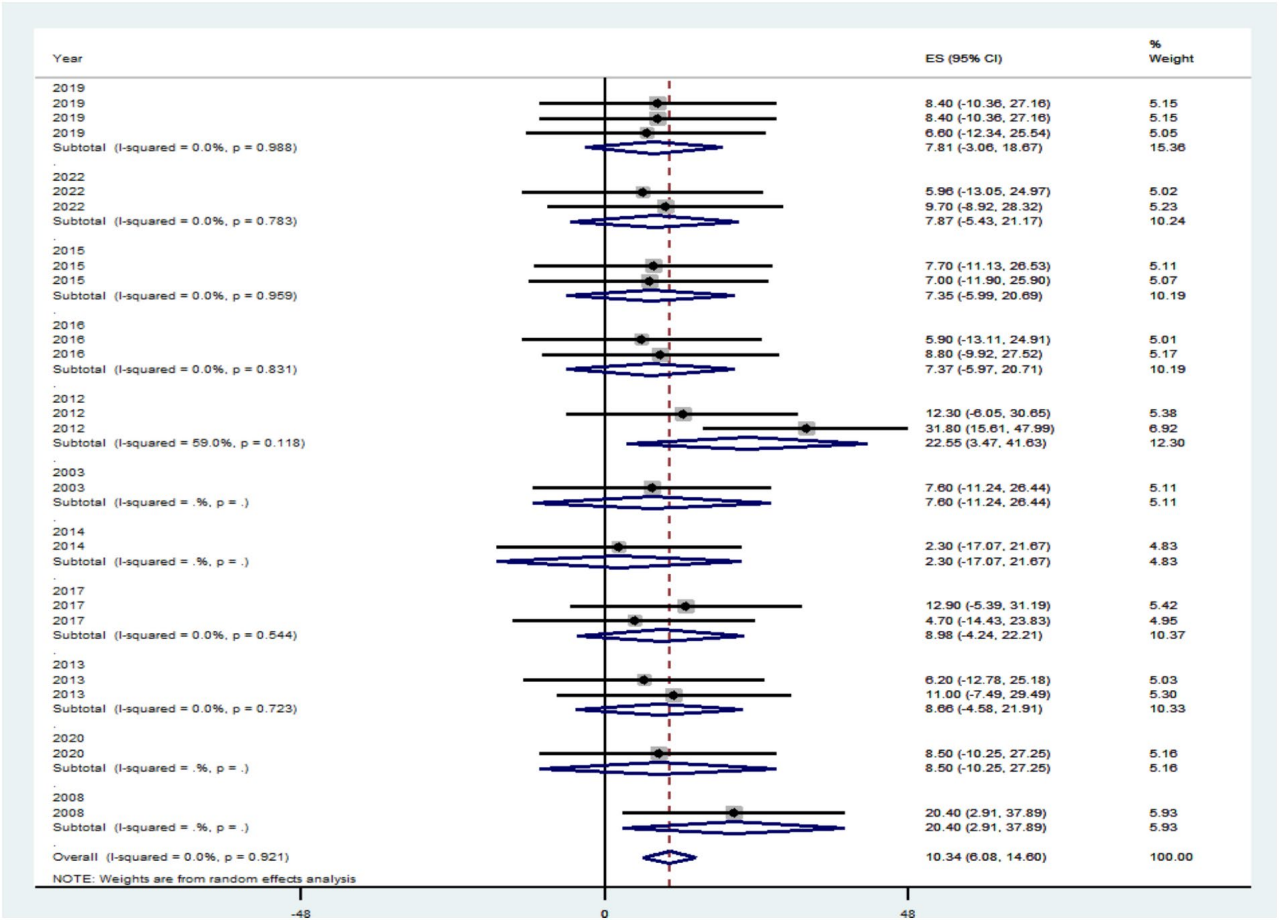
A graphical inspection of subgroup analysis from cross-sectional studies by study year.

In comparison to research articles by country (7.79%), the pooled level HIV–HBV comorbidity prevalence was significantly higher in articles from Ethiopia with 95% CI (−3.07 to 18.66%) at *p* > 0.05, and 7.71% the pooled level HIV–HBV comorbidity prevalence was significantly higher in articles from Ghana with 95% CI (−5.60 to 21.03%) at *p* > 0.05 (Figure 7). Regarding study year, studies conducted <2014 had a significantly lower pooled level of HIV–HBV comorbidity prevalence [2.30% with a 95% CI (−17.07 to 21.67%)] than articles conducted in 2012 [22.55% with a 95% CI (3.47 to 41.63%)] at *p* > 0.05 (Figure 8).

**Figure 9:**
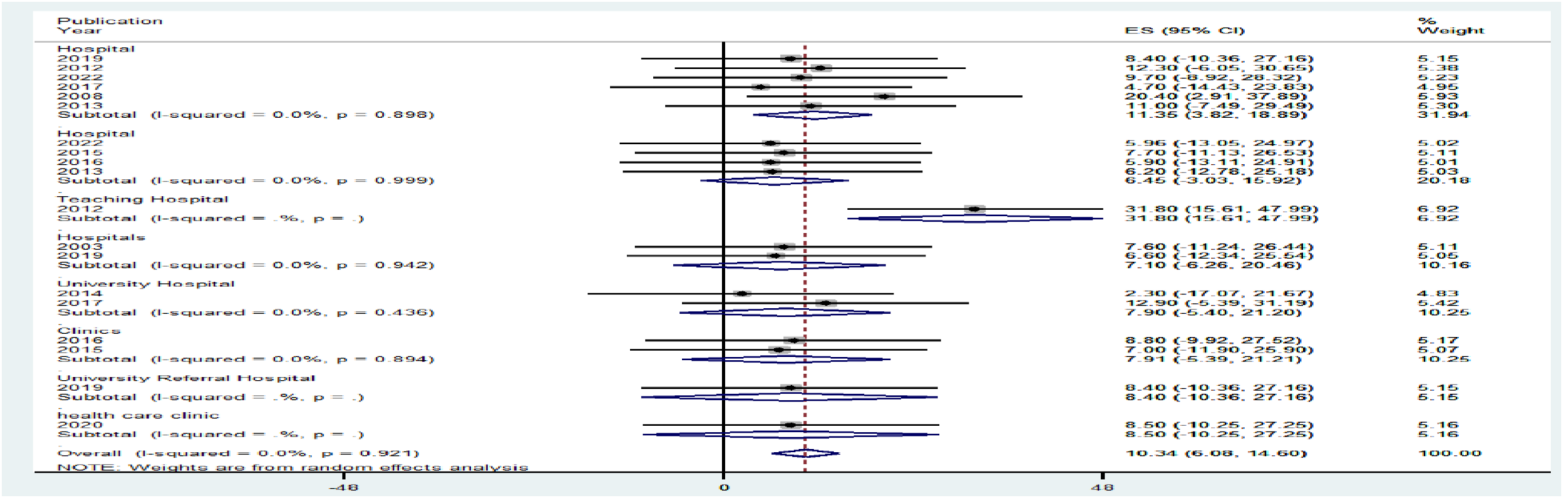
A graphical inspection of subgroup analysis from cross-sectional studies by study setting.

Regarding study setting, studies conducted in hospitals had a significantly lower pooled level of HIV–HBV comorbidity prevalence [6.53% with a 95% CI (−4.41 to 17.42%)] than articles conducted in teaching hospitals [31.80% with a 95% CI (15.61 to 47.99%)] at *p* > 0.05 (Figure 9).

### 5.4 Meta-regression

A meta-analysis with a significant amount of unexplained heterogeneity across the studies included in the review can use the statistical technique of meta-regression. It seeks to determine whether differences in study characteristics (methodological diversity) account for heterogeneity. This only works for meta-analyses that use a random-effects model.

However, the results of the regression analysis showed that there was no statistically significant association between heterogeneity in the prevalence of PLHIV–HBV comorbidity and sample size (*p* = 0.169), which could be interpreted as sample size, study period, study design, and study setting of comorbid prevalence were not identified as the causes of heterogeneity (Table 4).

**Table 4:** Prevalence and burden of HBV co-morbidity among people living with HIV of meta-regression from cross-sectional studies.

	Coef.	Std. Err	*z*	*p* > IzI	[95% conf. interval]
Year	−1.140165	0.8324519	−1.37	0.171	−2.771741 to 0.4914104
_Cons	2309.343	1678.632	1.38	0.169	−980.7156 to 5599.401

### 5.5 Sensitivity analysis

**Figure 10:**
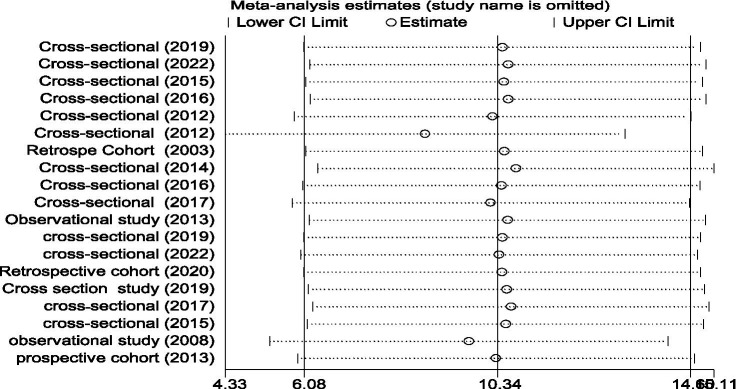
A graphical inspection of sensitivity from cross-sectional studies by country.

There are no studies outside the confidence bound, meaning that these two studies are outliers and have unequal influence on the pooled proportion (Figure 10).

### 5.6 Assessment of publication bias

#### 5.6.1 Funnel plot assessment of publication bias

A funnel plot was inspected graphically to determine whether it was symmetrical, with the horizontal axis showing the effect estimates from individual studies and the vertical axis reflecting the standard error of the effect estimate. Studies with large effect sizes were dispersed at the top of the funnel plot, whereas studies with small effect sizes were at the bottom.

**Figure 11:**
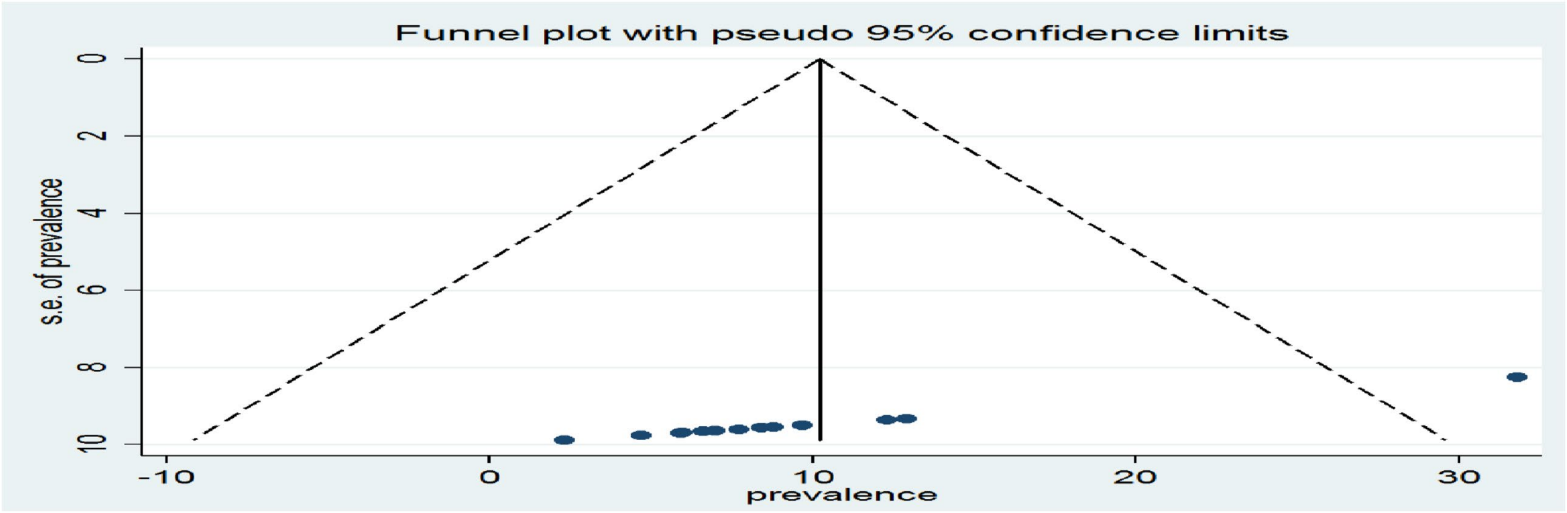
A graphical inspection of publication using a funnel plot of effect sizes versus the standard error of the effect sizes of cross-sectional studies.

The plot’s outcome resembled an inverted funnel with symmetry, indicating that there was no publication bias (Figure 11).

#### 5.6.2 Egger’s test of publication bias

Moreover, Egger’s test for small-study effects was also performed but was unable to show evidence of the existence of publication bias at *p* = 0.00, which is >0.05 and statistically not significant (Table 5).

**Table 5:** Egger’s test output assessment of publication bias of prevalence and burden of HBV co-morbidity among people living with HIV using cross-sectional studies.

Std-EFF	Coef.	Std. Err	*t*	*p* > ItI	[95% conf. interval]
Slope	180.7972	1.175675	153.78	0.000	178.2095 to 183.3848
Bias	−18.02103	0.1240732	−145.25	0.000	−18.29411 to 17.74795

#### 5.6.3 Trim and fill assessment for publication bias treatment

**Figure 12:**
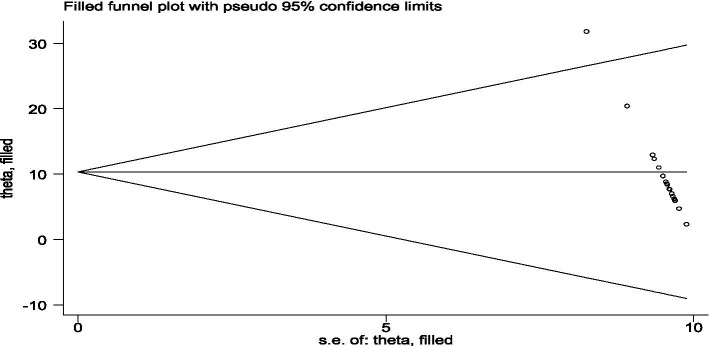
A graphical inspection of publication bias treatment using trim and fill assessment from cross-sectional studies.

The trim and fill method is a statistical technique used to detect and adjust for publication bias in meta-analysis. This method aims to identify and correct funnel plot asymmetry, which is often indicative of publication bias. Here is a detailed overview of the method, which starts by “trimming” or removing the smaller studies from one side of the funnel plot that contributes to the asymmetry. This is done iteratively until the funnel plot appears symmetric, filling once the plot is symmetric. The method “fills” in the missing studies by imputing mirror images of the trimmed studies around the estimated true effect size and finally reanalyzing. The filled studies are then added back into the meta-analysis, and the effect size is recomputed to obtain an adjusted estimate that accounts for the publication bias. Figure 12 shows all study articles were within the funnel plot and concentrated at the center of the funnel plot.

## 6 Discussion

The health transition has intensified recently against the backdrop of an evolving HIV treatment and prevention landscape, worsening the disease burden of HIV patients in a cycle of major societal developments like urbanization throughout the world. The broad appearance of the HBV epidemic as a result of the HIV-positive population’s general aging is one of the consequences, particularly for the young and middle-aged populations due to the early age of infection. As a result, our goal in writing this review is to provide an overview of all available data on HIV–HBV comorbidity worldwide, having previously examined inconsistent findings from multiple independent studies.

Although the prevalence of PLHIV–HBV comorbidity varies from country to country and study to study, in this review, the combined prevalence of PLHIV–HBV comorbidity was found to be the pooled prevalence of HIV–HBV comorbidity that was found to be 10.21 and 11.05% from cross-sectional and cohort studies, respectively. The result was higher than that of a previous meta-analysis (7.4%) (25, 26). This percentage is less than the findings from Cote d’Ivoire (31.8%), Nigeria (12.3%), and Cameroon (12.9%) but higher than those from other studies. The studies from all countries that were used to compare against this review were the findings from individual studies. As a result, the conclusions may have been slightly exaggerated, and these may not be the best explanations for the variations in the results.

Adult ART patients with a family history of HBV were 8.83 (2.56–30.5) times more at risk for infection with hepatitis B than those without a family history of HBV. This was consistent with the individual research findings in Addis Ababa, Ethiopia (27) and Dessie Referral and Kemise General Hospitals in northeastern Ethiopia (28). This might be due to genetic susceptibility, viral exposure, environmental factors, and lack of vaccination contributing to infection.

The pooled estimate of the reviewed literature showed that patients with ART who had multiple sexual partners were 7.08 (2.29–21.95) times at higher risk of infection with hepatitis B than those who had not had multiple sexual partners. This was consistent with the individual research findings in Addis Ababa, Ethiopia (27), and in Rome (29). This might be due to increased exposure, higher viral loads, trauma, bleeding, and increased STIs.

The pooled estimate of the reviewed literature showed that ART patients with a history of surgical procedures were 4.6 (1.8–11.6) times more at risk for infection with hepatitis B than those who had no history of surgical procedures. This was consistent with the individual research findings study in Uganda (30) and Hawassa City, Southern Ethiopia (31). This is because surgical procedures, particularly those involving invasive procedures, increase the likelihood of exposure to the surgeon’s blood, which can contain the virus. This exposure can occur through various means, such as needlestick injuries, cuts, or contact with other blood vessels during the procedure.

Those who had previous opportunistic infections were 5.2 (1.1–23.2) times at higher risk of hepatitis B infection than those who had not previously had opportunistic infections. This was consistent with the individual research findings of Debre Tabor Hospital in South Gondar, Ethiopia (32), China (33), and Maryland (34). Previous opportunistic infection is a risk factor for HBV infection because it weakens the immune system, making individuals more susceptible to infections.

The pooled estimate of the reviewed literature showed that ART patients with a CD4 count <200 cells/μL were 3.54 (1.12–11.21) times more at risk for infection with hepatitis B than those who had a CD4 count >500 cells/μL. This was consistent with the individual research findings in Tigray, Northern Ethiopia (35). This might be due to the higher incidence of mild liver disease and lower rates of spontaneous clearance of HBV, which enable the virus to establish chronic infection.

Those who had an AST > upper normal value (50 UI/mL) were 1.9 (1.02–3.6) times at higher risk for hepatitis B infection than those who had an AST < upper normal value (50 UI/mL). This result was consistent with the individual research findings in Japan (36), Spain (37), and Brazil (38). The reason for AST levels being lower in individuals with AST < upper normal value (50 IU/mL) compared to those with AST > upper normal value (50 IU/mL) is not explicitly stated in the provided sources. However, it can be inferred that the difference in AST levels is likely due to various factors, such as age, body mass index (BMI), and waist-to-hip ratio (WHR), which are known to influence AST levels.

The pooled estimate of the reviewed literature showed that patients with ART who had a recent alcohol abuse history were 1.7 (1.2–2.3) times at higher risk of infection with hepatitis B than those who had not. This result was consistent with the individual research findings in northern Portugal (39), China (40), and the United States (41). This might be due to the suppressed immune response, increased HBV replication, weakened immune responses, and causes of oxidative stress in the liver.

The pooled estimate of the reviewed literature showed that ART patients who had a recent history of abuse with injection drugs were 1.6 (1.1–2.4) times riskier for infection with hepatitis B than those who had no recent history of abuse with injection drugs. This was consistent with the individual research findings in Zanzibar (42) and San Francisco (43). The reason for the higher risk of hepatitis B virus (HBV) infection among those with a recent history of injection drug abuse is due to the sharing of contaminated injection equipment and unprotected sexual contact.

The pooled estimate of the reviewed literature showed that ART patients who had experience in tattooing on the body were 4.34 (1.21–15.58) times more at risk for infection with hepatitis B than those who had experience in tattooing on the body. This result was consistent with the individual research findings study with a systematic review and meta-analysis (44). This might be due to contaminated tattoo equipment and inks, an increasing number of tattoos, and tattooing in non-professional settings.

ART patients with a viral load ≥1,000 copies/mL were 5.53 (2.34–13.1) times more at risk of infection with hepatitis B than those who had a viral load <1,000 copies/mL. This result was consistent with the individual research findings in Johannesburg (45). This might be due to the correlation between high viral loads and the risk of developing advanced liver disease, such as cirrhosis and hepatocellular carcinoma (HCC).

Those ART patients who had HAART with lamivudine prescribed and used ART during the previous 6 months were 50% more preventive for infection of hepatitis B than those who had no HAART with lamivudine prescribed and used ART during the previous 6 months. This was consistent with individual research findings from different parts of the world (26, 46, 47). This might be HAART regimens containing lamivudine or tenofovir that appear to provide prophylaxis against hepatitis B virus (HBV) infection in HIV-infected individuals.

To adequately address the needs of patients with PLHIV in whom HBV and its risk factors coexist, we propose a research agenda to facilitate HIV–HBV care integration. This agenda focuses on research at the population and individual levels and includes an epidemiological, behavioral, and health systems focus. The study also addresses the call for focus in four main areas: defining the burden of HBV among patients with PLHIV who are on ART, understanding the impact of prevalence risk factors, evaluating effective and efficient care strategies at individual and health systems levels, and evaluating cost-effective prevention strategies.

Saving the lives of patients with PLHIV but then losing them prematurely to HBV would be disastrous. Providing HBV care as part of existing and functioning HIV care systems could be logistically simple and inexpensive but requires an evidence-based minimum package for HBV prevention, screening, and management that is appropriate for PLHIV. Many of the health system interventions that were used to scale up ART in resource-poor countries, such as standardized treatment protocols and task-shifting, can facilitate the effective management of the hepatitis B virus.

## 7 Limitations of the review

There are some important limitations, such as although the review’s researchers did their best to include all relevant information, there may still be certain pieces of literature that authors choose not to publish. The studies have enrolled samples from clinical settings. Findings from such samples usually provide higher estimates of the magnitude of comorbidities. Different countries have different HIV and HBV profiles. The variations in the findings across countries may be due to these differences in country profiles. We have used different methods to assess the causes of heterogeneity, but it remained unresolved. Furthermore, despite using extreme caution when searching, including and omitting publications, the pooled estimate could be affected by these practices. We specifically sought to summarize the burden of HBV among PLHIV who are on HAART. Therefore, the literature search and subsequent review may have missed data from articles that did not focus on these HBVs. The exclusion of qualitative studies from the review and the inclusion of articles published only in the English language were considered.

## Data Availability

The raw data supporting the conclusions of this article will be made available by the authors, without undue reservation.
